# Mesenchymal Stem Cells Ameliorate Hepatic Ischemia/Reperfusion Injury via Inhibition of Neutrophil Recruitment

**DOI:** 10.1155/2018/7283703

**Published:** 2018-12-03

**Authors:** Shihui Li, Xu Zheng, Hui Li, Jun Zheng, Xiaolong Chen, Wei Liu, Yan Tai, Yingcai Zhang, Genshu Wang, Yang Yang

**Affiliations:** ^1^Department of Hepatic Surgery and Liver transplantation Center of the Third Affiliated Hospital, Organ Transplantation Institute, Sun Yat-sen University, Organ Transplantation Research Center of Guangdong Province, Guangzhou, Guangdong 510630, China; ^2^Clinical Immunology Laboratory, Department of Rheumatology & Immunology, The First Affiliated Hospital of USTC, Hefei, Anhui 230001, China; ^3^Guangdong Provincial Key Laboratory of Liver Disease Research, The Third Affiliated Hospital, Sun Yat-sen University, Guangzhou, Guangdong 510630, China

## Abstract

Ischemia/reperfusion injury (IRI) remains a major problem in organ transplantation, which represents the main cause of graft dysfunction posttransplantation. Hepatic IRI is characterized by an excessive inflammatory response within the liver. Mesenchymal stem cells (MSCs) have been shown to be immunomodulatory cells and have the therapeutic action on IRI in several organs. However, the mechanism of regulatory effect of MSCs on IRI remains unclear. In the present study, we examined the impact of MSCs on hepatic inflammatory response such as neutrophil influx and liver damage in a rat model of 70% hepatic IRI. Treatment with MSCs protected rat against hepatic IRI, with significantly decreased serum levels of liver enzymes, attenuated hepatic neutrophil infiltration, reduced expression of apoptosis-associated proteins, and ameliorated liver pathological injury. MSCs also significantly enhanced the intracellular activation of p38 MAPK phosphorylation, which led to decreased expression of CXCR2 on the surface of neutrophils. In addition, MSCs significantly diminished neutrophil chemoattractant CXCL2 production by inhibiting NF-*κ*B p65 phosphorylation in macrophages. These results demonstrate that MSCs significantly ameliorate hepatic IRI predominantly through its inhibitory effect on hepatic neutrophil migration and infiltration.

## 1. Introduction

Liver transplantation is one of the most efficient life-saving treatments for various end-stage hepatic diseases [1]. However, hepatic IRI remains a major problem in this clinical setting. IRI affects liver viability that usually leads to delayed recovery or even loss of graft function in liver transplantation [2]. Moreover, IRI directly correlates to graft rejection, which causes up to 10% of early transplant failures and also leads to a higher incidence of chronic rejection [3]. Hepatic IRI also plays a critical role in donor shortage due to the higher sensitivity to IRI of clinical marginal liver donors.

During hepatic IRI, liver damages are caused largely during the reperfusion period, when an excessive innate immune response is triggered by blood reperfusion [4]. The mechanisms of reperfusion-induced pathological and functional alterations are not fully understood and under intensive investigation [5]. The results of previous studies are complicated, but all showed that significant inflammatory components are involved. Neutrophils are considered central factors in the events leading to injury after reperfusion [6].

MSCs exert a variety of immune-regulatory functions on cells within both adaptive and innate immunity, which make MSC therapy more efficient than single-agent drug therapies [7]. MSCs could suppress proliferation and functions of effector T cell [8] and enhance generation of regulatory T cells [9]. MSCs also inhibit B cell, natural killer cells, and dendritic cell activation and proliferation [10–12]. In addition, MSCs could induce a shift from proinflammatory M1 to anti-inflammatory M2 macrophages [13]. Studies have rarely investigated the protective effects of MSCs in hepatic IRI and related mechanism of the beneficial effect of MSCs on ischemia reperfusion-injured tissues. None of them has investigated the interactions between MSCs and neutrophils during hepatic IRI, in spite of the key role of neutrophils in innate immune response-mediated tissue damage after reperfusion

In this study, we focus on the effects of MSCs on neutrophils which exert protective role on neutrophil-mediated tissue damage during hepatic IRI.

## 2. Materials and Methods

### 2.1. Ethics Statements

Male Sprague-Dawley (SD) rats weighing 80–100 g and 200–250 g from the Laboratory Animal Center of Sun Yat-sen University (Guangzhou, China) were used for all experiments. All animal experiments were carried out in accordance with the National Institutes of Health Guide for the Care and Use of Laboratory Animals (NIH Publication No. 8023, revised 1978). All of the experimental procedures were approved by the Sun Yat-sen University Ethics Committee.

### 2.2. Model of Hepatic Ischemia/Reperfusion Injury

The hepatic artery and portal vein to the left lateral and median lobes of male SD rats weighing 200–250 g were clamped for 60 min by clamp after being anesthetized. Reperfusion was initiated by removing the clamp. A total of 3 × 10^5^ MSCs at passages 3–5 suspended in 1 mL PBS or 1 mL PBS were injected via the portal vein, respectively, in the MSC + IRI group and the PBS group immediately after the removal of the clamp. The animals in the sham group underwent the same anesthesia, laparotomy, and injection procedures, except the interruption of blood supply to the liver lobes.

### 2.3. Isolation and Characterization of Rat MSCs

Rat MSCs were prepared from rat bone marrow cells as we previously described [14]. Briefly, bone marrow cells flushed from the femurs of male SD rats were collected and cultured in Dulbecco modified Eagle medium (DMEM; Gibco, Rockville, MD) supplemented with 10% fetal calf serum (FCS; Gibco), 2 ng/mL basic fibroblast growth factor (b-FGF; Gibco), and 1% penicillin/streptomycin (P/S; Gibco), at 200,000 cells/cm^2^ at 37°C in 5% CO_2_. The nonadherent cells were removed after 24 hours, and the adherent cells were harvested by trypsinization (0.05% trypsin) when reaching 80% confluence. The immunophenotypes of these cells were persistently positive for CD29, CD44, and CD90 but negative for CD34, CD45, and CD11b for more than three passages. To determine their multilineage differentiation potential, MSCs at passages 3–5 were subjected to adipogenesis differentiation and osteogenesis differentiation media (Gibco) according to the manufacturer's instructions.

### 2.4. Isolation of Neutrophils

Blood was collected from male SD rats and diluted in 1 × HBSS (Hanks balanced salt solution; Gibco) at 1 : 1 ratio and layered over Percoll plus (GE Healthcare) in a centrifuge tube. The red cell pellet that contains the neutrophils and erythrocytes was collected and suspended in 5 mL HBSS after centrifugation. Erythrocytes were removed by hypotonic lysis using ACK Lysis Buffer (TBD sciences, Tianjing). Purified neutrophils were suspended in HBSS and were kept on ice until needed.

### 2.5. Coculture Experiment

Neutrophils or macrophages were placed in the lower chamber and stimulated for 12 h with 10 ng/mL of LPS in the presence or absence of MSCs in the upper chamber at a 10 : 1 ratio by using the transwell system (0.3 mm pore size membrane; Corning, Cambridge, MA).

### 2.6. Flow Cytometry

For phenotypic analysis of the cell surface marker expression, cells were harvested, then incubated with respective fluorescent antibodies for 30 minutes at 4°C, washed twice, and resuspended in 300 *μ*L PBS. For intracellular staining, cells were fixed by formaldehyde and permeabilized by ice-cold methanol before immunostaining. Fluorescent antibodies included phycoerythrin- (PE-) conjugated anti-rat CD29, CD44, CD90, CD34, CD45, and CD11b (eBioscience, San Diego, CA), allophycocyanin- (APC-) conjugated anti-rat CXCR2 (R&D Systems, Minneapolis, MN), Alexa fluor 647-conjugated anti-rat phospho-p38 MAPK, and phospho-NF-*κ*B p65 (Cell Signaling Technology, Danvers, MA). The results were processed using FlowJo software (Tree Star, Inc).

### 2.7. Analysis of Relative Gene Expression Using Real-Time Quantitative Reverse Transcription PCR

Real-time quantitative reverse transcription PCR (qRT-PCR) analysis was performed 12 h after the liver or cells were treated. Total RNA was isolated using Trizol reagent (Invitrogen, Carlsbad, CA) according to the manufacturer's instructions. Total RNA (1 mg) extracted from each sample was reverse transcribed to cDNA in a 20 *μ*L reaction mixture using an RT reagent kit (Roche) according to the manufacturer's directions. Real-time qRT-PCR was performed for the candidate genes and for GAPDH as the internal control. Quantitative real-time PCR was performed in a Light Cycler Real-Time PCR machine using Roche Fast Start Universal SYBR Green Master (Rox). The primers are shown in Table 1. The specificity of the PCR products was verified by melting curve analysis. Each sample was analyzed in triplicate.

### 2.8. Western Blot Analysis

Equal amounts of protein from each sample were separated by 12% SDS-PAGE and transferred to PVDF membranes (Millipore, Boston, MA). The proteins were then incubated overnight at 4°C with primary antibodies against rat Bad, Fas, Cleaved caspase 3, phospho-p38 MAPK, and *β*-actin (Cell Signaling Technology); following a 30 min wash, the membranes were incubated with a secondary antibody conjugated to HRP for 1 h at room temperature. After being washed for 30 min, the membranes were visualized by enhanced chemiluminescence (ECL; Millipore, Billerica, MA) and recorded by FluorChem M (Protein Simple, San Jose, California).

### 2.9. Measurement of Inflammatory Cytokines and Chemokine

The concentration of IL-2, IL-4, IL-6, IL-10, and CXCL2 in the liver and cell culture supernatants was quantitatively measured by enzyme-linked immunosorbent assay (ELISA) (EIAab Science), according to the manufacturer's instructions. In order to detect the inflammatory cytokines and chemokine in the liver, the homogenate made from the liver tissue was mixed with precooled PBS (100 mg/500 *μ*L), and the supernatant was collected after centrifugation (500g, 5 min) for further ELISA testing.

### 2.10. Histopathology and Immunohistochemistry

The harvested liver tissues were fixed in 4% paraformaldehyde, embedded in paraffin, cut into 4 *μ*m sections, and stained with hematoxylin and eosin. For immunohistochemistry, endogenous peroxidases, nonspecific binding sites were blocked using 0.3% H_2_O_2_ and Goat Serum, respectively. Then, sections were incubated with an anti-rat MPO Ab at 4°C overnight. Subsequently, the Catalyzed Signal Amplification System (DAKO, K1500) was used for staining. Then, the slides were counterstained with hematoxylin. Liver sections were evaluated blindly by counting labeled cells in 10 high-power fields.

### 2.11. TUNEL Stain

TUNEL stain was performed to detect the apoptosis of liver tissue sections by using TUNEL kit (KeyGEN, China) according to the instructions. Apoptotic cells were observed under fluorescence microscope, and the nuclei of the positive cells were presented as bright green.

### 2.12. Prussian Blue Staining of MSCs Labeled with SPIO

MSCs were labeled with Ferumoxytol (Southeast University, Nanjing, China), as previously described [15]. Briefly, polyamine poly-l-lysine (PLL) (Sigma, USA) was used as the transfection agent. PLL (10.0 *μ*g/mL) was mixed with Ferumoxytol (500 *μ*g/mL) for 60 min at room temperature on a rotating shaker. MSCs of passages 3–5 were cultured with the medium that contained the Ferumoxytol-PLL complex (50 *μ*g/mL Ferumoxytol) for 24 h. After being incubated with the Ferumoxytol-PLL complex, the MSCs were washed three times to remove excessive contrast agent. For Prussian blue staining, which indicates the presence of iron, the coverslip samples were fixed with 4% paraformaldehyde for 30 min, washed, incubated for another 30 min with 2% potassium ferrocyanide in 6% hydrochloric acid, washed again, and counterstained with nuclear fast red.

### 2.13. Statistical Analysis

All values are expressed as the mean ± SD. Data were analyzed with an unpaired two-tailed Student's *t*-test. *P* < 0.05 was considered statistically significant.

## 3. Results

### 3.1. Characterization of Rat MSCs

**Figure 1:**
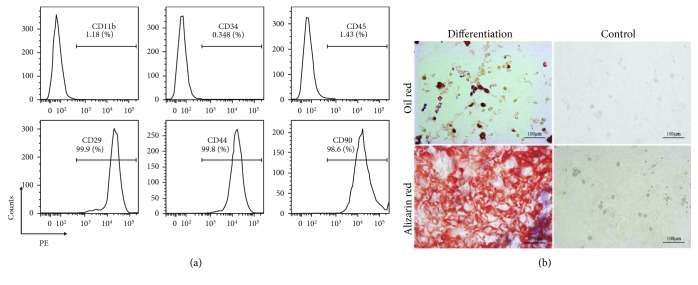
Characterization of rat bone marrow derived-MSCs. (a) Immunophenotype of rat MSCs. Cells were harvested at passage 3, labeled with the antibodies specific for the indicated surface antigens or negative controls, and analyzed by flow cytometry. The numbers in the panels represent the mean fluorescence intensity of the cells expressing each marker. (b) Rat MSCs at passage 3 that were induced to differentiate into adipocytes and osteoblasts. Cells stain positive for oil with oil red staining and for calcium with alizarin red solution, respectively. Original magnification, ×200.

Rat MSCs at passage 3 were analyzed for the expression of cell surface molecules by flow cytometry. The MSCs expressed CD29, CD44, and CD90 but did not express hematopoietic cell markers such as CD45, CD34, and CD11b (Figure 1(a)) which have been previously reported.

In addition, the differentiation capacity of MSCs was also examined. Rat MSCs could be differentiated into osteoblasts and adipocytes, which represent classical mesenchymal lineage cells (Figure 1(b)).

### 3.2. MSCs Reduce the Release of Liver Enzymes and Improve the Histopathologic Changes of the Livers in IRI

**Figure 2:**
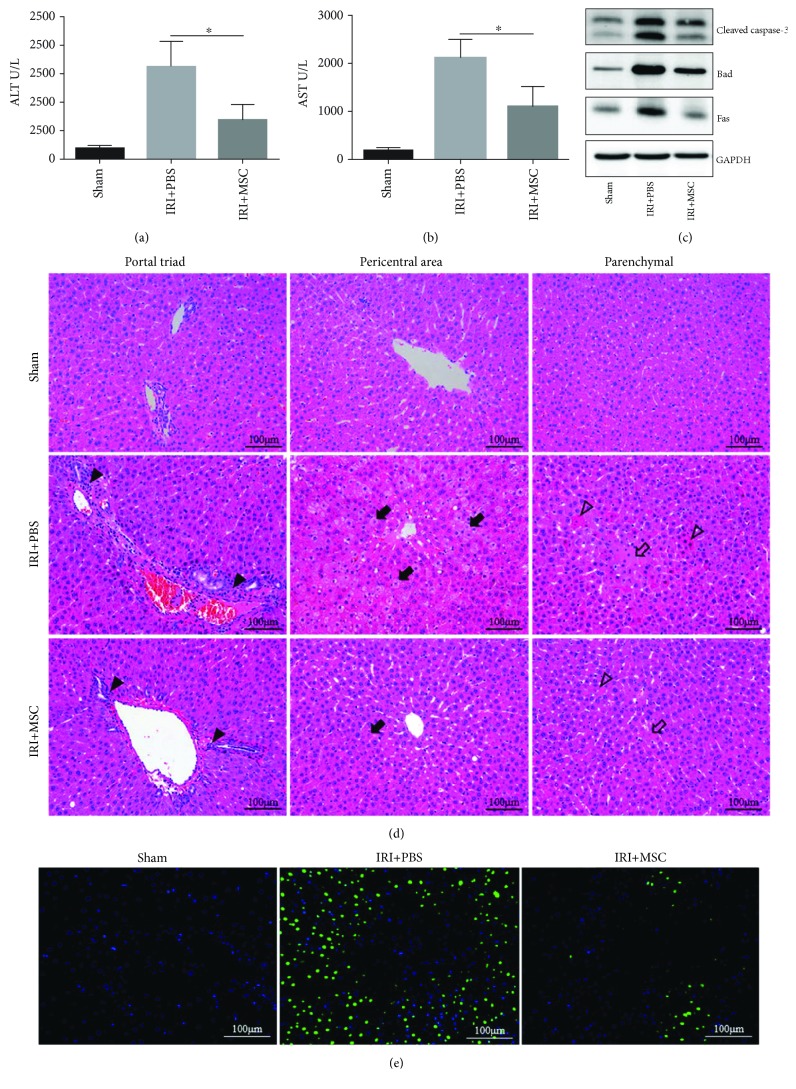
Rat MSCs reduced the release of liver enzymes and improved the histopathologic changes of livers in IRI. Male SD rats were randomized into sham, IRI + PBS, and IRI + MSC groups. Samples of each group were collected 12 h postreperfusion. (a, b) As markers for hepatic injury, serum levels of ALT and AST were determined. (c) The expression levels of various proapoptotic proteins including Cleaved caspase-3, Bad, and Fas in the I/R liver lobes were determined by Western blot analysis. (d) Representative images of hematoxylin and eosin-stained sections of liver tissues were shown. Inflammatory cell accumulation (solid black triangles), hepatocellular swelling (solid black arrows), necrosis (hollow arrows), and acidophilic degeneration (hollow triangles) were observed. (e) TUNEL staining of apoptosis cells (bright green) in liver tissues. Data are mean ± SD; ^∗^*p* < 0.05. Original magnification, ×200.

To investigate the protective effect of MSCs in rat liver IRI model, we measured serum ALT (Figure 2(a)) and AST (Figure 2(b)) after 12 h of I/R. ALT and AST levels in rats treated with MSCs were significantly reduced compared to those in rats treated with PBS injections. At the same time, decreased expression levels of various proapoptotic proteins in IRI liver lobes including Cleaved caspase-3, Bad, and Fas were found in response to MSC treatment compared to the control group (Figure 2(c)). In addition, the histopathologic injury of the liver including portal inflammation, hepatocyte swelling, cytoplasm rarefaction, and coagulative necrosis was alleviated in the MSC-treated group. After 12 h of reperfusion, the IRI + PBS group had significantly more inflammatory cell accumulation (solid black triangles) in the portal area compared to the IRI + MSC group. The count of swelling hepatocyte (solid black arrows) in the IRI + PBS group is much more than that in the IRI + MSC group, as well as the degree of cytoplasm rarefaction. The IRI + PBS group also had markedly higher hepatocellular necrosis (hollow arrows) and acidophilic degeneration (hollow triangles) compared to the IRI + MSC group (Figure 2(d)). Furthermore, the TUNEL stain of the liver showed that the number of apoptosis cells (bright green) in the MSC treatment group was rarer than that in the IRI + PBS group (Figure 2(e)).

### 3.3. MSCs Affect Cytokine Expression and Reduce Neutrophil Infiltration

**Figure 3:**
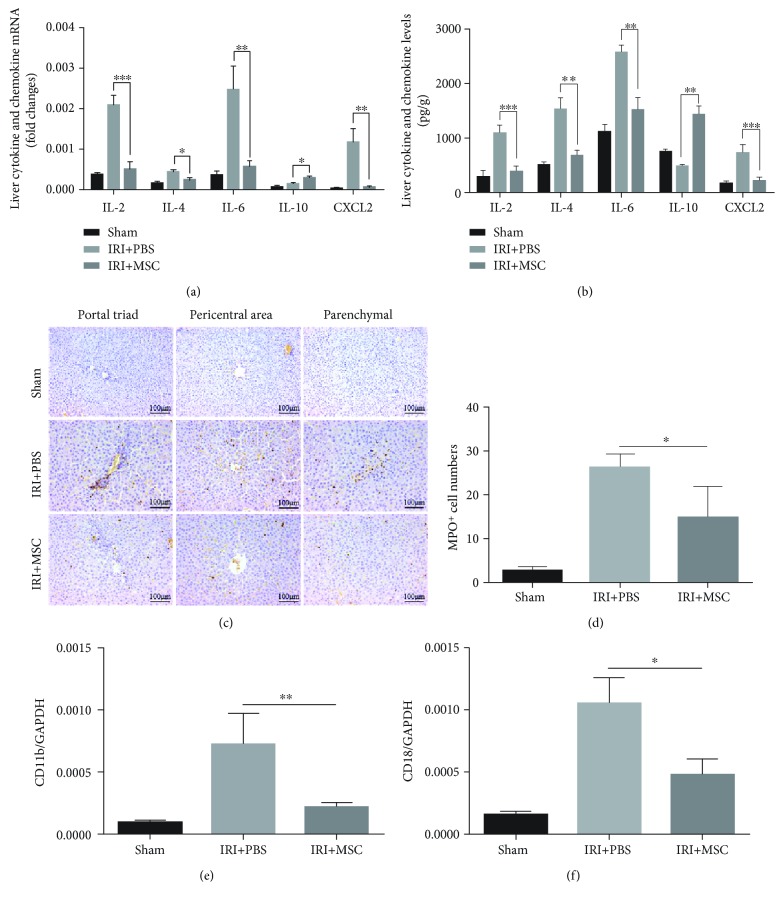
MSCs affect cytokine expression and reduce neutrophil infiltration. (a, e, f) The relative mRNA levels of IL-2, IL-4, IL-6, IL-10, CXCL2, CD11b, and CD18 genes in I/R lobes were detected by qRT-PCR. Data were normalized to glyceraldehyde-3-phosphate dehydrogenase gene expression. (b) The concentration of IL-2, IL-4, IL-6, IL-10, and CXCL2 in I/R lobes was measured by ELISA. (c) Representative images of immunohistochemistry-stained sections of MPO^+^ cells of liver tissues were shown. (d) The mean frequency of hepatic MPO^+^ cells in 10 high-power fields was calculated. Data are mean ± SD. ^∗^*p* < 0.05, ^∗∗^*p* < 0.01, ^∗∗∗^*p* < 0.001. Original magnification, ×200.

Following I/R injury, there were significant increases in proinflammatory cytokines IL-2, IL-4, and IL-6 and chemokine CXCL2 at both mRNA (Figure 3(a)) and protein (Figure 3(b)) levels in the liver. Treatment with MSCs markedly attenuated I/R-stimulated CXCL2 and proinflammatory cytokine aforementioned expression in the liver. Furthermore, the immunohistochemical analysis showed distinct neutrophil infiltration compared to the sham group, and the cell infiltration decreased significantly after MSC therapy (Figures 3(c) and 3(d)). The change of levels of the CD11b/CD18 mRNA also indicated that MSC injection could reduce neutrophil infiltration (Figures 3(e) and 3(f)).

### 3.4. MSCs Accumulated in the Injured Liver Lobes in the Model of Hepatic IRI

**Figure 4:**
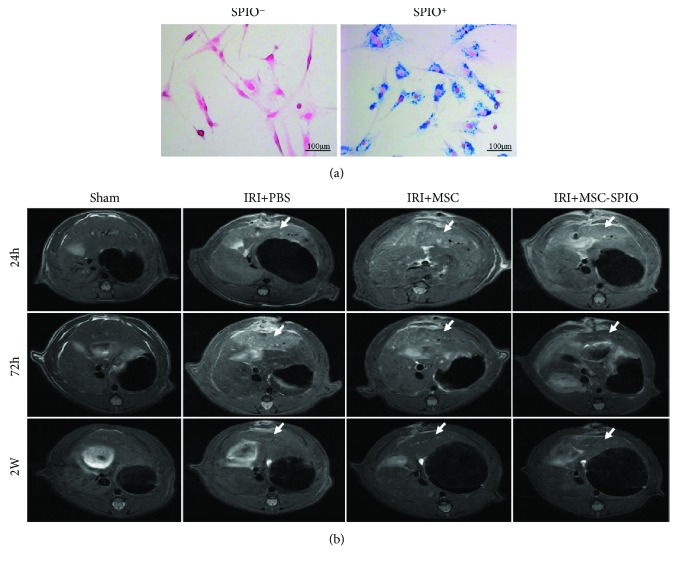
MSCs accumulated in injured liver lobes in the model of hepatic IRI. (a) SPIO-labeled MSCs were positive for Prussian blue staining. Blue particles were observed in the cytoplasm of SPIO^+^ cells. (b) Male SD rats were randomized into sham, IRI + PBS, IRI + MSC, and IRI + MSC-SPIO groups. Representative images of MRI scanning on the T2 sequence of I/R lobes in each group were shown after 24 h, 72 h, and 2 w of reperfusion. The liver lobes experienced IRI were pointed out by white arrows. Original magnification, ×200.

MSCs were known to migrate or dock preferentially to injured sites [16]. This phenomenon could facilitate the paracrine of MSCs considered a principal mechanism in MSC therapy [17]. Thus, we labeled MSCs with SPIO and tracked the accumulation of MSCs at 24 h, 72 h, and 2 w postinjection in the model of hepatic IRI. SPIO-labeled MSCs stained with Prussian blue showed blue particles in the cytoplasm in contrast with unlabeled cells in which no blue particles were observed, and the labeling ratio was approximately 100% (Figure 4(a)). The signal intensity of the injured liver lobe increased on the T2 sequence in both the IRI + PBS group and the IRI + MSC group that implied the degree of inflammation of the lobes, but the signal of the IRI + MSC group was significantly lower than that of the IRI + PBS group after 72 h, which means MSC injection could protect the liver during IRI. In the IRI + MSC-SPIO group, in which MSCs were labeled with SPIO, the signal intensity of IRI lobes was significantly lower than that of others 72 h after reperfusion indicated the recruitment of MSCs in these areas. In addition, the signal intensity gradually approached close to normal on day 14 (Figure 4(b)).

### 3.5. MSCs Attenuate Neutrophil Recruitment via Downregulation of CXCR2

**Figure 5:**
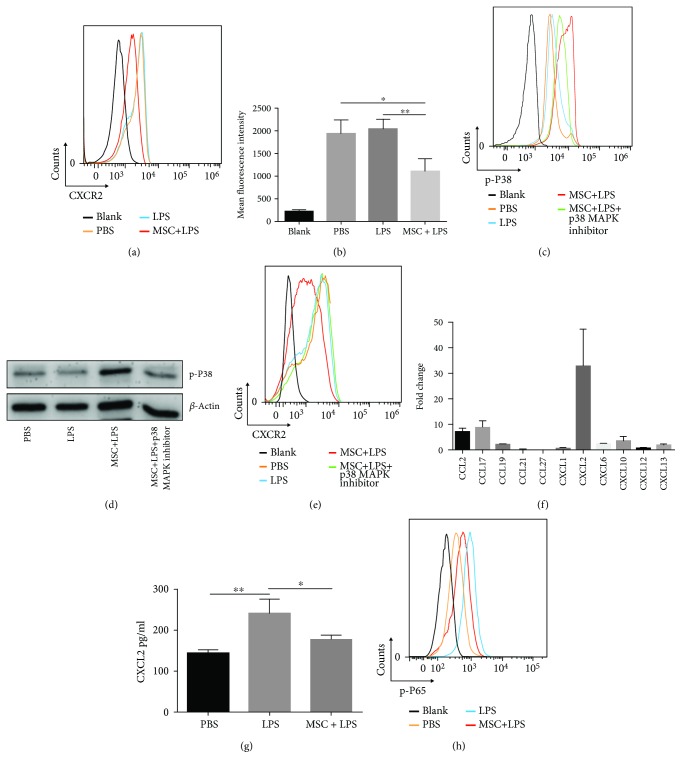
MSCs attenuate neutrophil recruitment via hindering CXCL2/CXCR2 signaling. (a, e) Surface expression of CXCR2 on neutrophils was detected by flow cytometry analysis. (b) The mean fluorescence intensity of CXCR2 on the surface of neutrophils was recorded. (c) The levels of p38 MAPK phosphorylation in neutrophils were detected by flow cytometry analysis, and (d) the consequences were verified by Western blot analysis. (f) The expression levels of mRNA of various chemokines involved in hepatic IRI were analyzed by qRT-PCR. Fold change represents the expression of each chemokine in I/R liver lobes 12 h postreperfusion compared with normal liver. (g) Macrophages were cultured in the presence or absence of MSCs for 12 h. The concentration of CXCL2 in the supernatant was measured by ELISA. (h) The levels of NF-*κ*B p65 phosphorylation in macrophages were detected by flow cytometry analysis. Data are mean ± SD. ^∗^*p* < 0.05, ^∗∗^*p* < 0.01, ^∗∗∗^*p* < 0.001. p-P38: phospho-p38 MAPK; p-P65: phospho-NF-*κ*B p65.

To study the mechanism(s) underlying the decrease of infiltration of neutrophils in IRI liver, we examined the surface expression of CXCR2, an important chemokine receptor that mediates neutrophil recruitment. As shown in Figures 5(a) and 5(b), MSC treatment significantly downregulated the surface expression of CXCR2 on neutrophils. Further, we found that the inhibitory effect on CXCR2 expression was caused by enhanced intracellular activation of p38 MAPK phosphorylation (Figures 5(c) and 5(d)) and has been blocked by p38 MAPK inhibitor (Figure 5(e)).

### 3.6. MSCs Inhibit Macrophage CXCL2 Expression via Attenuation of NF-*κ*B p65 Phosphorylation

As shown in Figure 5(f), the CXCL2 mRNA expression level was almost 30 times higher in I/R injured liver lobes and exhibited the most significant change among the tested chemokines. Injection of MSCs significantly attenuated CXCL2 mRNA expression (Figure 3(a)) and protein production (Figure 3(b)). In vitro, these changes of CXCL2 expression (Figure 5(g)) were correlated with the phosphorylation of NF-*κ*B p65 in macrophages. MSCs inhibited the activation of NF-*κ*B p65 of macrophages (Figure 5(h)).

## 4. Discussion

Hepatic IRI occurs in diverse clinical situations, such as hepatic resection, liver transplantation, shock, and trauma. In particular, in liver transplantation, IRI is the main cause of morbidity and mortality due to graft rejection [18]. Previous studies have shown that liver damage is mainly caused during reperfusion period, and neutrophils are considered central factors in the events leading to injury after reperfusion [19]. MSCs represent a promising candidate for liver cell therapy because of their immunomodulation of both adaptive and innate immunity [20]. So far, few studies have examined the effects of MSCs on hepatic IRI [21]. In this study, we investigate the roles of MSCs on hepatic inflammatory response, neutrophil recruitment, and liver tissue damage in a rat model of partial hepatic IRI. We reported that injected MSCs accumulated in damaged liver lobes and effectively protected rat against hepatic IRI, with significantly decreased serum levels of liver enzymes, attenuated hepatic neutrophil infiltration, reduced expression of apoptosis-associated proteins, and ameliorated liver pathological changes. We further demonstrated that MSCs significantly inhibited IRI-stimulated overexpression and release of neutrophil chemoattractant CXCL2 through attenuation of NF-*κ*B p65 activation and substantially attenuated neutrophil chemotaxis via downregulation of CXCR2 expression by increasing of p38 MAPK phosphorylation in neutrophils.

As shown in previous studies, hepatic IRI should be considered as an innate immunity-dominated inflammation response [22]. Liver damage is driven by a complex set of leukocytes, including natural killer cells, natural killer T cells, dendritic cells, neutrophils, and eosinophils. Neutrophils, the largest circulating fraction of leukocytes, arrive at the injury site first and play the crucial role in liver injury. It has been reported that the extent of neutrophil sequestration in patients with ischemic or alcoholic liver disease correlates strongly with disease severity [23], while depletion of neutrophils before hepatic insult can limit tissue injury in animal experiments [24]. In the present study, after 12 h of reperfusion in the IRI rats, histopathologic examination showed markedly hepatocellular injury including swelling, apoptosis, and necrosis, which was consistent with high levels of AST and ALT in the serum. The pathology was observed by the concurrent recruitment of neutrophils in the liver. Treatment with MSCs significantly attenuated I/R-induced influx of neutrophils into the liver, thus ameliorating liver injury. We further confirmed the MSC homing phenomenon by using MRI tracking technology. As with similar other studies [25–27], MSCs docked preferentially to injured sites. In consideration of the paracrine and regeneration mechanism of MSCs in cell therapy, their ability of trafficking to particular tissues deserves further research [28].

We next examined the underlying mechanisms by which MSCs attenuate neutrophil accumulation in the I/R liver. As mentioned, CXCR2 signaling is an important chemokine axis that regulates neutrophil release from the bone marrow [29] and recruitment from the circulation into the site of inflammation [30]. Depletion of CXCR2 on neutrophils could significantly alleviate organ inflammation due to the reduction of neutrophil infiltration [31]. We found that MSC treatment significantly downregulates CXCR2 expression on neutrophils, thus attenuating neutrophil chemotaxis toward the I/R liver lobe. It has been shown that the chemoattractant CXCL2 plays a key role in neutrophil recruitment. The expression of CXCL2 protein in the ischemic lobes was increased hundred- to thousandfold over control [32]. Furthermore, the expression of CXCL2 was much earlier than neutrophil accumulation, which suggests that CXCL2 may be involved in the initial recruitment of neutrophils to the ischemic lobe [33]. Our findings are lined with previous studies that I/R resulted in markedly increased CXCL2 expression in the liver at both mRNA and protein levels. Interestingly, the MSC treatment significantly reduced I/R-stimulated CXCL2 production. Taking into account both of the impacts, MSCs attenuate neutrophil chemotaxis by hindering CXCL2/CXCR2 signaling.

Prior to neutrophil infiltration, the hypoxic-ischemic damage of resident liver cells during ischemic phase results in the release of endogenous molecules named danger-associated molecular patterns (DAMPs), which can be recognized by pattern recognition receptors expressed on innate immune cells thus causing neutrophil gathering [34]. Toll-like receptors (TLRs) play important roles among these DAMP receptors including retinoic acid-inducible gene I-like receptors, nucleotide-binding oligomerization domain-like receptors, and C-type lectin receptors. Notably, the activation of MAPK and NF-*κ*B signaling pathways was enrolled during the recognition of DAMPs [35]. Previous studies showed that the increase of phosphorylation levels of the p38 MAPK was related to neutrophil chemotaxis [36]. In this study, we found activation of p38 MAPK phosphorylation in neutrophils upon treatment with MSCs, which led to decreased expression of CXCR2 on the cell surface. Furthermore, the inhibitory effect of MSCs on CXCR2 expression can be blocked by p38 MAPK inhibitor. Macrophages were considered the main source of CXCL2 in liver injury [37]. We then examined the effect of MSCs on NF-*κ*B p65 and MARK p38 activation in macrophages. MSCs substantially inhibited NF-*κ*B p65 phosphorylation, thereby attenuating CXCL2 expression and production in macrophages. These results suggest a synergy between the affection of MSCs on neutrophils and macrophages to alleviate hepatic IRI.

In conclusion, we have demonstrated that MSCs may represent a potential therapeutic strategy to alleviate hepatic ischemia/reperfusion injuries. The effects of MSCs were found, for the first time, due to the inhibition ability of neutrophil chemotaxis via NF-*κ*B p65 and MAPK p38 signaling pathways. The results of this study offer a new insight into the mechanisms responsible for MSC-mediated inhibition, a protective manner, which may promote the future clinical application of MSCs in liver transplantation.

## Figures and Tables

**Table 1:** Gene names and primer sequences used for quantitative RT-PCR.

Gene name	Primers used	
	Sense (5′ to 3′)	Antisense (5′ to 3′)
IL2	GCAGGCCACAGAATTGAAAC	CCAGCGTCTTCCAAGTGAA
IL4	GTCACTGACTGTAGAGAGCTATTG	CTGTCGTTACATCCGTGGATAC
IL6	GAAGTTAGAGTCACAGAAGGAGTG	GTTTGCCGAGTAGACCTCATAG
IL10	AGTGGAGCAGGTGAAGAATG	GAGTGTCACGTAGGCTTCTATG
CD11b	GAGCACCATCTGGGACATAAA	GGCATCAGAGTCCACATCAA
CD18	CCAGTAACGTAGTCCAGCTTATC	CATAGGTGACTTTCAGGGTGTC
CCL2	GTCTCAGCCAGATGCAGTTAAT	CTGCTGGTGATTCTCTTGTAGTT
CCL17	GTGCTGCCTGGACTACTT	CTTCCCTGGACAGTCTCAAA
CCL19	GCCTTCCGCTACCTTCTTATC	GTCTTCGGATGATGCGTTCT
CCL21	GGCCGTCCCTTTCTTCTATG	AGTCCTGCTGTCTCCTTCT
CCL27	CTCCAACAAGCCAGAGACTAAG	CTCCAACAAGCCAGAGACTAAG
CXCL1	GCACCCAAACCGAAGTCATA	GGGACACCCTTTAGCATCTTT
CXCL2	GACAGAAGTCATAGCCACTCTT	GCCTTGCCTTTGTTCAGTATC
CXCL6	GCTCAAGCTGCTCCTTTCT	GCAGGGATCACCTCCAAATTA
CXCL10	AAGCGGTGAGCCAAAGAA	CAGGAGAAACAGGGACAGTTAG
CXCL12	GGGAAGGGAAACGGAGAAAG	CTCACCACACACACATCACTAA
CXCL13	CCAAGCTCCAGTGAGTAAGAAA	AAGATTCCGAGCAGGGATTAAG
GAPDH	ACTCCCATTCTTCCACCTTTG	CCCTGTTGCTGTAGCCATATT

## Data Availability

The data used to support the findings of this study are included within the article.
